# Simultaneous anterior cruciate ligament reconstruction and implant-mediated guided growth to correct genu valgum in skeletally immature patients

**DOI:** 10.1016/j.jisako.2023.03.003

**Published:** 2023-03-17

**Authors:** Peter D. Fabricant, Danielle E. Chipman, Nicolas Pascual-Leone, Joshua Bram, Damiano Salvato, Daniel W. Green

**Affiliations:** Department of Pediatric Orthopaedic Surgery, Hospital for Special Surgery, 535 E 70th Street, New York, NY, 10021, USA

**Keywords:** Anterior cruciate ligament, Coronal plane angular deformity, Implant-mediated guided growth

## Abstract

**Objectives:**

Adolescents with anterior cruciate ligament (ACL) tears can present with concomitant lower extremity coronal plane angular deformity (CPAD) that both predispose to injury as well as may increase the risk of graft rupture following ACL reconstruction (ACLR). The goal of this study was to examine the safety and efficacy of concomitant ACLR with implant-mediated guided growth (IMGG) compared to isolated IMGG procedures in paediatric and adolescent patients.

**Methods:**

Operative records of all paediatric and adolescent patients (age ≤ 18 years) that underwent simultaneous ACLR and IMGG by one of two paediatric orthopaedic surgeons between 2015 and 2021 were retrospectively reviewed. A comparison cohort of isolated IMGG patients was identified and matched based on bone age within one year, sex, laterality, and fixation type (i.e. transphyseal screw vs. tension band plate and screw construct). Pre- and post-operative mechanical axis deviation (MAD), angular axis deviation (AAD), lateral distal femoral angle (LDFA), and medial proximal tibial angle (MPTA) were recorded.

**Results:**

A total of 9 participants who underwent concomitant ACLR and IMGG (ACLR + IMGG) were identified, with 7 of these participants meeting the final inclusion criteria. The participants had a median age of 12.7 (IQR = 12.1 – 14.2) years and median bone age of 13.0 (IQR = 12.0 – 14.0) years. Of the 7 participants that underwent ACLR and IMGG, 3 underwent a modified MacIntosh procedure with ITB autograft, 2 received quadriceps tendon autograft, and 1 underwent hamstring autograft reconstruction. There were no significant differences in the amount of correction obtained between ACLR + IMGG and matched IMGG subjects with respect to any measurement variable (MAD difference: p = 0.47, AAD difference: p = 0.58, LDFA difference: p = 0.27, MPTA difference: p = 0.20). There were also no significant differences in alignment variables per unit time between cohorts (MAD/month: p = 0.62, AAD/month = 0.80, LDFA/month = 0.27, MPTA/month = 0.20).

**Conclusion:**

The results of the current study indicate that concomitant ACLR and lower extremity CPAD correction is a safe approach to treat CPAD concomitantly with ACLR in young patients who present with an acute ACL tear. Furthermore, one can expect reliable correction of CPAD after combined ACLR and IMGG, no different than the correction obtained in the setting of IMGG alone.

**Level of evidence:**

III.

## Introduction

In recent years, there has been a marked increase in paediatric anterior cruciate ligament (ACL) injuries [1]. Many of these patients also present with concomitant lower extremity coronal plane angular deformity (CPAD) [2]. Valgus malalignment is particularly detrimental as it can potentially increase strain on the ACL, leading to a predisposition to graft rupture after initial ACL reconstruction (ACLR) [3–9].

One option to address lower extremity CPAD is through implant-mediated guided growth (IMGG), which has been shown to reliably correct coronal malalignment [10–14]. IMGG is a less invasive technique to correct lower extremity malalignment than a traditional tibial osteotomy, which is often the technique of choice to achieve correction of malalignment in adults [3,15,16]. More recent literature points to the possibility of providing correction in concomitant ACLR and IMGG [10, 17]. However, to our knowledge, no study has directly compared alignment correction rates or surgical outcomes between patients undergoing simultaneous ACLR with IMGG procedures versus patients that underwent isolated IMGG.

Therefore, the aim of this study was to compare the correction of alignment obtained with concomitant ACLR and IMGG to isolated IMGG procedures in paediatric patients. We hypothesized that ACLR with concomitant IMGG would provide appropriate, safe correction of lower extremity CPAD, and that the degree of correction would not differ significantly from isolated IMGG procedures.

## Methods

### Participants

This is a retrospective cohort study of paediatric patients who underwent concomitant ACLR and IMGG between 2015 and 2021 by one of two fellowship-trained paediatric sports surgeons. After institutional review board approval, patients less than or equal to 18 years of age that underwent ACLR and IMGG were identified using Current Procedural Terminology (CPT) codes (ACLR = 29888, IMGG = 27475 and 27485). A total of 15 participants met these inclusion criteria. Participants were excluded if they lacked both pre- and post-operative full-length lower extremity standing radiographs.

### Comparison cohort

The comparison cohort consisted of paediatric patients that underwent isolated IMGG between 2015 and 2021 and were collected using the same CPT codes (IMGG = 27475 and 27485). The cohorts were then matched based on skeletal age within one year, sex, laterality, and IMGG fixation type (i.e. transphyseal screw vs. tension band plate and screw construct).

### Radiographic measurements

Pre- and post-operative measurements were made on full-length lower extremity standing radiographs for the ipsilateral knee. Post-operative measurements were made on the radiograph closest to their implant removal surgery date with the hardware still in place. Measurements included mechanical axis deviation (MAD), angular axis deviation (AAD), lateral distal femoral angle (LDFA), and medial proximal tibial angle (MPTA) [18–24]. Post-operative values were subtracted from pre-operative values to determine the differences in alignment variables. Correction per unit time was also assessed, which was calculated as the difference in axis variables divided by the time in months between the last post-operative radiograph and the initial date of surgery.

### Statistics

Descriptive statistics were run on all patients and are reported as medians with interquartile ranges (IQR). Using IBM SPSS Version 22 for Windows, Wilcoxon signed-rank test was used to compare pre- and post-operative measurements for patients who had concomitant ACLR and IMGG. Mann–Whitney U tests were then used to compare continuous variables. A two-tailed p-value of ≤ 0.05 was used to determine statistical significance.

## Results

A total of 9 subjects underwent concomitant ACLR and IMGG, with 7 of these participants included in the final analysis (ACLR + IMGG). One participant was excluded due to lateral distal femoral growth arrest associated with ACLR that required a formal epiphysiodesis, and one participant failed to have a comparison cohort match due to age.

The remaining 7 participants that underwent concomitant ACLR and IMGG had a median age of 12.7 (IQR = 12.1–14.2) years and median bone age of 13.0 (IQR = 12.0–14.0) years (Table 1). Of the 7 participants, 5 (71%) were male, 4 (57%) participants underwent bilateral IMGG, and 6 (86%) participants had IMGG implant fixation using both screws and plates. Three (43%) participants underwent a modified MacIntosh procedure with ITB autograft, 3 (43%) received quadriceps tendon autograft, and 1 (14%) underwent hamstring autograft reconstruction (Table 2). The median clinical follow-up was 1.6 (IQR = 1.2–2.9) years.

At the time of the most recent follow-up, hardware had been removed in 5 (71%) cases, having been maintained for a median 0.9 (IQR = 0.8–1.2) years. The technique used for ACLR varied in the 7 participants with 2 (29%) undergoing all-epiphyseal ACLR, 2 (29%) undergoing complete transphyseal ACLR, and 3 (43%) undergoing extra-articular reconstructions (modified MacIntosh ACLR). No patients that underwent simultaneous ACLR and IMGG had complications including graft injury, wound infection, or deep vein thrombosis/pulmonary embolism, however, one patient had a post-operative complication of arthrofibrosis necessitating reoperation.

**Fig. 1.**
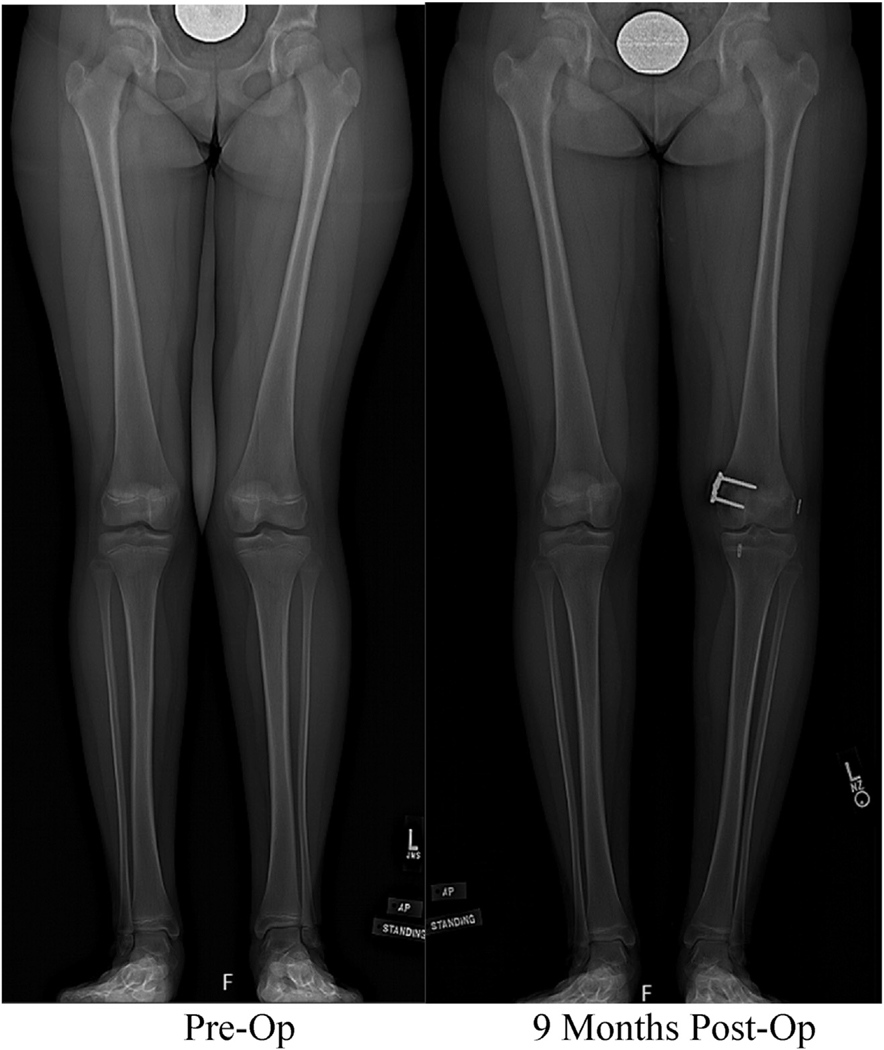
Pre-operative and post-operative full-length lower extremity standing radiographs of a 11-year-old female.

Measurements on full-length lower extremity radiographs were made on 5 patients in each cohort, excluding 2 patients in each cohort because they did not have their hardware removed because they reached skeletal maturity. Pre- and post-operative deformity measurements on full-length lower extremity radiographs demonstrated significant correction in MAD, AAD, and LDFA for participants that underwent concomitant ACLR and IMGG procedures (MAD: p = 0.02, AAD: p = 0.00, LDFA: p = 0.02, MPTA: 0.23) (Table 3) (Fig. 1).

These 7 participants were matched to a similar cohort of patients who underwent isolated IMGG. There were no significant differences in the pre-operative to post-operative difference in axis variables between groups (MAD difference: p = 0.47, AAD difference: p = 0.58, LDFA difference: p = 0.27, MPTA difference: p = 0.20) (Table 4). The median change in MAD per unit time for participants that underwent concomitant ACLR and IMGG was 1.4 (IQR = 1.0–3.1) mm/month per month and 2.0 (IQR = −1.4 – 3.3) mm/month for participants that underwent isolated IMGG (p = 0.62) (Table 5). The median change in AAD per unit time for patients with concomitant procedures was −0.4° (IQR = −0.8 – 0.3) per month and −0.5° (IQR = −1.1 – 0.3) for isolated IMGG (p = 0.80).

## Discussion

This study evaluated the safety and efficacy of concomitant ACLR and lower extremity CPAD correction in paediatric patients. This study demonstrates reliable correction of lower extremity CPAD after combined ACLR and IMGG among paediatric patients, no different than the correction obtained after isolated IMGG. This suggests that performing IMGG at the time of ACLR provides adequate lower extremity CPAD correction. Past work has demonstrated that coronal malalignment increases forces on the reconstructed ACL and predisposes to ACL tear [16,25,26]. This study validates the ability to safely address both diagnoses at once.

IMGG has a long history of being an effective treatment of pathologic genu valgum. In 1949, Blount et al. described the mechanism behind IMGG using stainless steel staples at the distal femoral and proximal tibial epiphyses [27]. They observed that CPAD can be corrected using staples without risk of growth arrest after staple removal [27]. Stevens et al. further described IMGG for idiopathic genu valgum in 1999 [28]. They reviewed 152 knees that underwent IMGG for genu valgum until skeletal maturity was achieved and found that IMGG was both safe and effective, leading to improvements in anatomic alignment and clinical symptoms [28]. Stevens et al. reported that their indications for IMGG in treating genu valgum were 1) at least 2 years of growth remaining and 2) a mechanical axis that falls on the outer quadrant of the knee or beyond [28]. However, IMGG is contraindicated if there is a physeal bar or the patient is not able to comply with routine follow-up.

There is unfortunately little literature on the topic of combined ACLR and IMGG. O’Brien et al. retrospectively evaluated eight skeletally immature patients who underwent concomitant transphyseal ACLR and hemi-epiphysiodesis, demonstrating significant improvements in alignment [17]. However, they did not evaluate a similar cohort of patients undergoing isolated guided growth, making it difficult to understand the adequacy of correction achieved with concomitant procedures. Ellsworth et al. studied IMGG performed with surgery for other knee pathologies including five patients with ACLR [10]. They similarly reported appropriate correction rates for simultaneous ACLR and IMGG procedures. While this study also showed significant corrections in alignment following combined ACLR and IMGG, we were further able to demonstrate that these corrections were similar to those obtained for isolated IMGG.

Past literature on isolated IMGG for coronal malalignment has shown mean LDFA correction rates of 0.32–0.40°/month [17,29]. In this series, median pre-operative LDFA was 84.5° (normal 87.0°). Utilizing mean correction rates for LDFA of 0.30–0.40°/month, an estimated 9–11 months of guided growth are required to achieve neutral alignment [17]. This corresponds to the observed total time of hardware implantation in the current study of 10.8 months.

Valgus malalignment is thought to be a risk factor for ACL graft rupture. Valgus malalignment can be either a non-modifiable structural valgus or a dynamic functional valgus, with patients sometimes presenting with both forms [4]. Biomechanical data has shown that increased valgus load increases the risk of ACL injury, particularly for female athletes [4,30–33]. In a cadaveric-based study, Withrow et al. demonstrated that a valgus knee moment increased the anteromedial strain on the ACL by 30% [5]. Price et al. reported that the inherent risk factor of anatomic valgus is increased by dynamic valgus movements, which inevitably leads to an increased incidence of ACL injuries [4]. Therefore, we believe if pathologic genu valgum is not corrected at the time of ACLR, the patient is at increased risk of graft rupture although we acknowledge that this had not been proven with an epidemiologic study [16,25,26].

One concern about performing ACLR and IMGG concomitantly with the addition of another procedure is the potential to further increase the already moderate risk of post-operative arthrofibrosis (typically defined by a loss of greater than 5° of extension or 10° of flexion) [34]. The reported rate in the literature of arthrofibrosis in the knee for paediatric and adolescent patients that undergo ACLR ranges from about 1.8% to 8.3% [35–38]. Su et al. conducted a retrospective case–control study to investigate the characteristics related to arthrofibrosis after paediatric ACLR and found that there were 20 cases (1.8%) of arthrofibrosis out of a total of 1121 ACLR patients (mean age = 14.5 years) [37]. Additionally, in 2017, Cruz et al. retrospectively reviewed 103 patients (mean age = 12.1 years) and found 2 cases (1.9%) of arthrofibrosis [36]. Common treatment options for arthrofibrosis are manipulation under anaesthesia, lysis of adhesions, and debridement of scar tissue. In this study, one patient that underwent concomitant ACLR and IMGG had a post-operative complication of arthrofibrosis. However, given the small sample size of this study, it is difficult to draw conclusions on the degree to which concomitant IMGG may have increased the risk of post-operative arthrofibrosis beyond that of ACLR alone.

There were several limitations in this study. First, this was a retrospective study with a small sample size due to the relative infrequency of this combined procedure. However, this population size is larger than those of previous studies on this topic, and our comparison cohort analysis allows one to better understand the adequacy of correction achieved after ACLR and IMGG compared to isolated IMGG [10,17]. Second, the included patients underwent several different types of ACLR, which may make comparison of correction and outcomes across these subgroups difficult. Lastly, while two patients had retained IMGG hardware at the time of the most recent follow-up and therefore final correction was not determined, correction per unit time was used to take into account the possibility of further correction in select patients. Future studies should evaluate ACLR and IMGG in a prospective, large series of patients to assess if alignment correction using this technique decreases graft failure rates.

## Conclusion

Concomitant ACLR and lower extremity malalignment is a novel approach to treat pathologic genu valgum in paediatric patients with an acute ACL tear to lower the likelihood of future graft rupture. Our study demonstrates appropriate correction of lower extremity alignment after combined ACLR and IMGG that was similar to patients undergoing isolated IMGG. This suggests that these procedures can be performed simultaneously and deliver appropriate correction of coronal malalignment.

## Figures and Tables

**Table 1:** Demographics of ACLR + IMGG group.

	Median	IQR
Age (years)	12.7	12.1–14.2
Bone Age (years)	13.0	12.0–14.0
BMI	24.10	16.2–25.6
Length of Follow-up (years)	1.6	1.2–2.9
Time IMGG Intact (years)	0.9	0.8–1.2

IMGG, Implant-Mediated Guided Growth; BMI, Body Mass Index.

**Table 2:** Surgical details of ACLR + IMGG group.

	n	%
**ACLR Graft Type**		
Modified MacIntosh with ITB Autograft	3	43
All-Epiphyseal with Quadriceps Autograft	2	29
Complete Transphyseal with Quadriceps Autograft	1	14
Complete Transphyseal with Hamstring Autograft	1	14
**Additional Procedures at Index Surgery**		
Meniscus Repair	3	43
ITB Tenodesis	2	29
Meniscectomy	2	29
**Second Surgeries**		
Hardware Removal	5	71
Lysis of Adhesions & Manipulation	1	14
**Return to Sport**		
Yes	6	86
No	1	14
**ACL Re-tears**		
No	7	100

ACLR, Anterior Cruciate Ligament Reconstruction; IMGG, Implant-Mediated Guided Growth; ITB, Iliotibial Band.

**Table 3:** Pre-operative and post-operative deformity measurements in participants that underwent concomitant ACLR and IMGG. Excluded participants were those that reached skeletal maturity by the latest follow-up (n = 5).

	Pre-operative	Post-operative	P-value
		
	Median (IQR)		α = 0.05
MAD (mm)^a^	−16.8 (−19.0–−5.9)	5.5 (1.4–17.4)	**0.02**
AAD (°)	8.3 (8.1–10.6)	2.5 (1.5–5.2)	**0.00**
LDFA (°)	84.5 (82.7–87.8)	90.0 (88.6–91.1)	**0.02**
MPTA (°)	90.0 (89.5–91.3)	89.5 (86.9–90.0)	0.23

MAD, Mechanical Axis Deviation; AAD, Angular Axis Deviation; LDFA, Lateral Distal Femoral Angle; MPTA, Medial Proximal Tibial Angle. Bold values are statistically significant (*p* ≤ 0.05).

a Negative values = valgus, positive values = varus.

**Table 4:** Differences (post-operative measurement - pre-operative measurement) in deformity measurements in participants that underwent concomitant ACLR and IMGG versus participants who underwent isolated IMGG. Excluded participants were those that reached skeletal maturity by the latest follow-up (included n = 5 per group).

	ACLR + IMGG	IMGG only	P-value
		
	Median (IQR)		α = 0.05
MAD difference (mm)	19.2 (12.6 – 32.5)	21.1 (−1.7 – 25.4)	0.47
AAD difference (°)	−6.5 (−7.7–−4.0)	−6.4 (−8.2 – 0.4)	0.58
LDFA difference (°)	5.7 (2.7 – 6.4)	4.4 (−2.1 – 4.8)	0.27
MPTA difference (°)	−0.5 (−4.4 – 0.6)	0.2 (−0.6 – 0.8)	0.20

ACLR, Anterior Cruciate Ligament Reconstruction; IMGG, Implant-Mediated Guided Growth; MAD, Mechanical Axis Deviation; AAD, Angular Axis Deviation; LDFA, Lateral Distal Femoral Angle; MPTA, Medial Proximal Tibial Angle.

**Table 5:** Deformity correction per unit time in participants that underwent concomitant ACLR and IMGG compared to isolated IMGG. Excluded participants were that reached skeletal maturity by the latest follow-up (included n = 5 per group).

	ACLR + IMGG	IMGG only	P-value
		
	Median (IQR)		α = 0.05
MAD (mm/month)	1.4 (1.0 – 3.1)	2.0 (−1.4 – 3.3)	0.62
AAD (°/month)	−0.4 (−0.8 – 0.3)	−0.5 (−1.1 – 0.3)	0.80
LDFA (°/month)	0.1 (0.03 – 0.07)	0.05 (−0.03 – 0.05)	0.27
MPTA (°/month)	−0.01 (−0.05 – 0.01)	0.00 (−0.01 – 0.01)	0.20

ACLR, Anterior Cruciate Ligament Reconstruction; IMGG, Implant-Mediated Guided Growth; MAD, Mechanical Axis Deviation; AAD, Angular Axis Deviation; LDFA, Lateral Distal Femoral Angle; MPTA, Medial Proximal Tibial Angle.

## What are the new findings?

- Anterior cruciate ligament reconstruction and lower extremity coronal plane angular deformity correction can safely be performed concomitantly.
- Concomitant anterior cruciate ligament reconstruction and lower extremity coronal plane angular deformity correction can effectively treat coronal plane angular deformity concomitantly with anterior cruciate ligament reconstruction in young patients who present with an acute anterior cruciate ligament tear.
- There is no difference in the correction obtained in the setting of implant-mediated guided growth alone compared to the correction of coronal plane angular deformity after combined anterior cruciate ligament reconstruction and implant-mediated guided growth.
